# Cinobufagin inhibits tumor growth by inducing intrinsic apoptosis through AKT signaling pathway in human nonsmall cell lung cancer cells

**DOI:** 10.18632/oncotarget.7898

**Published:** 2016-03-03

**Authors:** Guangxin Zhang, Chao Wang, Mei Sun, Jindong Li, Bin Wang, Chengyan Jin, Peiyan Hua, Ge Song, Yifan Zhang, Lisa L.H. Nguyen, Ranji Cui, Runhua Liu, Lizhong Wang, Xingyi Zhang

**Affiliations:** ^1^ Department of Thoracic Surgery, Second Hospital of Jilin University, Changchun, P.R. China; ^2^ Department of Genetics, University of Alabama at Birmingham, Birmingham, Alabama, USA; ^3^ Department of Integrative Endemic Area, Tongji Hospital of Huazhong University of Science and Technology, Wuhan, P.R. China; ^4^ Department of Pathology, Second Hospital of Jilin University, Changchun, P.R. China; ^5^ Jilin Provincial Key Laboratory on Molecular and Chemical Genetic, Second Hospital of Jilin University, Changchun, P.R. China

**Keywords:** cinobufagin, non-small cell lung cancer, apoptosis, reactive oxygen species, mitochondrial transmembrane potential

## Abstract

The cinobufagin (CB) has a broad spectrum of cytotoxicity to inhibit cell proliferation of various human cancer cell lines, but the molecular mechanisms still remain elusive. Here we observed that CB inhibited the cell proliferation and tumor growth, but induced cell cycle arrest and apoptosis in a dose-dependent manner in non-small cell lung cancer (NSCLC) cells. Treatment with CB significantly increased the reactive oxygen species but decreased the mitochondrial membrane potential in NSCLC cells. These effects were markedly blocked when the cells were pretreated with N-acetylcysteine, a specific reactive oxygen species inhibitor. Furthermore, treatment with CB induced the expression of BAX but reduced that of BCL-2, BCL-XL and MCL-1, leading to an activation of caspase-3, chromatin condensation and DNA degradation in order to induce programmed cell death in NSCLC cells. In addition, treatment with CB reduced the expressions of p-AKT^T308^ and p-AKT^S473^ and inhibited the AKT/mTOR signaling pathway in NSCLC cells in a time-dependent manner. Our results suggest that CB inhibits tumor growth by inducing intrinsic apoptosis through the AKT signaling pathway in NSCLC cells.

## INTRODUCTION

Lung cancer is the leading cause of cancer deaths around the world [1]. Non-small cell lung cancer (NSCLC) accounts for at least 75% of all lung cancer cases [2] and the majority of them are diagnosed at advanced stages and are inoperable [3]. To data, targeted therapy represents a meaningful advance in the treatment of NSCLC, but platinum-based chemotherapy still plays a major role in the treatment of patients with advanced NSCLC. Platinum drugs, including cisplatin, gemcitabine, docetaxel, and paclitaxel, can induce DNA adducts through an apoptotic cascade of signaling transduction pathways in cancer cells [4]. However, platinum-based chemotherapy represents a therapeutic challenge in NSCLC patients who are resistant to platinum drugs after long-term treatment, leading to little improvement in patient survival coupled with the unsatisfactory results of substantial toxicities. Even with the addition of target-therapy, the median survival in most metastatic NSCLC patients is less than one year [5]. While the need to develop better strategies to overcome this challenge is urgent, current resistance-modulating approaches have generally not proven clinically useful in NSCLC patients [6]. Hence, development of novel agents as an alternative approach may overcome the resistance to platinum-based chemotherapy in NSCLC patients.

Numerous traditional Chinese medicines have been observed to present potent anti-cancer activities and have attracted a great deal of interest as potential candidates for the development of novel anti-cancer drugs [7]. For example, astragalus, a Chinese herb, has been shown to improve effectiveness of platinum-based chemotherapy in NSCLC patients [8]. Recently, we have screened several compounds derived from Chinese medicines for anti-cancer agents and our identified candidates induced anti-cancer effects through a mitochondria-mediated apoptosis in NSCLC cells, such as alantolactone [9], cepharanthine [10], dracorhodin [11]. Specifically, the ethanol extract from the Chinese herb Chan Su showed a strong cytogenotoxic effect on a human NSCLC cell line A549 [12]. Bufadienolides cinobufagin is one of the primary chemical compounds of Chan Su [13, 14], which is a cardiotonic steroid isolated from the skin and parotid venom glands of the toad *Bufo gargarizans Cantor* [15]. Chan Su has been used as a significant anti-cancer agent, enhancing the life quality of cancer patients [16]. Cinobufagin (CB) has also been shown to have significant anti-cancer effects in several cancers, including liver cancer [17], cervical cancer [18], and prostate cancer [19], but its anti-cancer mechanism still remains elusive. Although CB as a member of the cardiac glycoside family inhibits Na+/K+-ATPase activity [20], CB also emerged recently as a key inhibitor of cell proliferation without serious side effects in cancer cells [21]. Thus, CB appears to be an alternative anti-cancer drug for NSCLC patients who are resistant to platinum-based chemotherapy. In the present study, we aim to determine the anti-cancer effect of CB and its anti-cancer mechanism in NSCLC cells.

## RESULTS

### CB dose-dependently inhibits the tumor growth of human NSCLC cell lines

**Figure 1:**
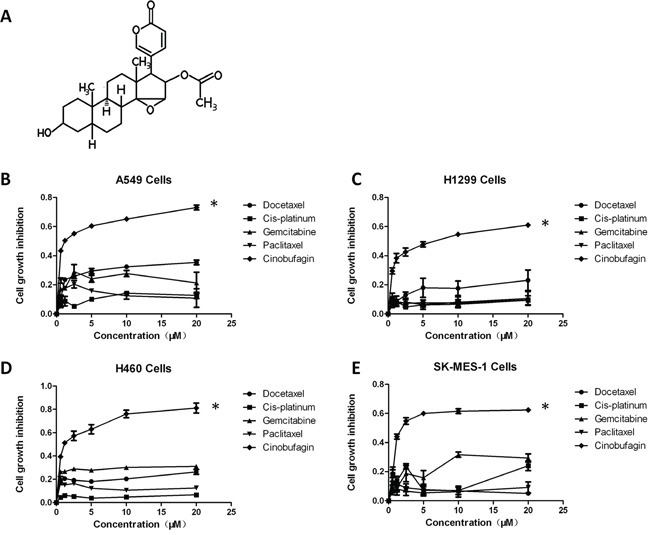
The effects of CB on cell viability in human NSCLC cell lines **A.** chemical structure of CB. **B-E.** effects of CB and platinum drugs on the growth inhibition in four NSCLC cell lines by the MTT assay. Cells were treated with different concentrations of CB for 24 hours. Data are presented as mean ± SD of triplicates. All * *p* < 0.0001, CB vs. platinum drugs, Fisher's PLSD test. All experiments were repeated three times.

CB is one of the bufadienolides (resibufogenin, cinobufagin, and bufalin) isolated from the Chinese traditional medicine Chan Su (Figure 1A). Early studies have revealed that CB has a broad spectrum of cytotoxicity to inhibit cell proliferation of various human cancer cell lines [19, 22, 23]. To determine whether CB effectively inhibits the growth of human NSCLC cells, we selected four NSCLC cell lines, including A549 (lung adenocarcinoma), H1299 (lung adenocarcinoma), H460 (lung large cell carcinoma), and SK-MES-1 (lung squamous cell carcinoma), which harbor different genetic mutations involved in diverse signaling pathways, such as EGFR, RAF, and mTOR signaling pathways. These four NSCLC cell lines were treated with varying concentrations of CB in comparison with platinum drugs, including cisplatin, gemcitabine, docetaxel, and paclitaxel. Since the half maximal inhibitory concentration (IC_50_) values vary in different cancer cells [22], a gradient concentration (0, 0.6, 1.2, 2.5, 5, 10, and 20 μM) of CB and platinum drugs was used for treatment in all cell lines. Treatment with CB or an individual platinum drug for 24 hours reduced the cell viability in a dose-dependent manner on the four NSCLC cell lines (Figure 1B-1E). A 40-50% inhibitive efficacy was identified in cells treated with less than a 2 μM concentration of CB. In treatments with the same drug concentration, there were more significant anti-proliferative effects of CB compared with those of platinum drugs (Figure 1B-1E), suggesting a higher anti-cancer efficacy of CB in NSCLC cells.

**Figure 2:**
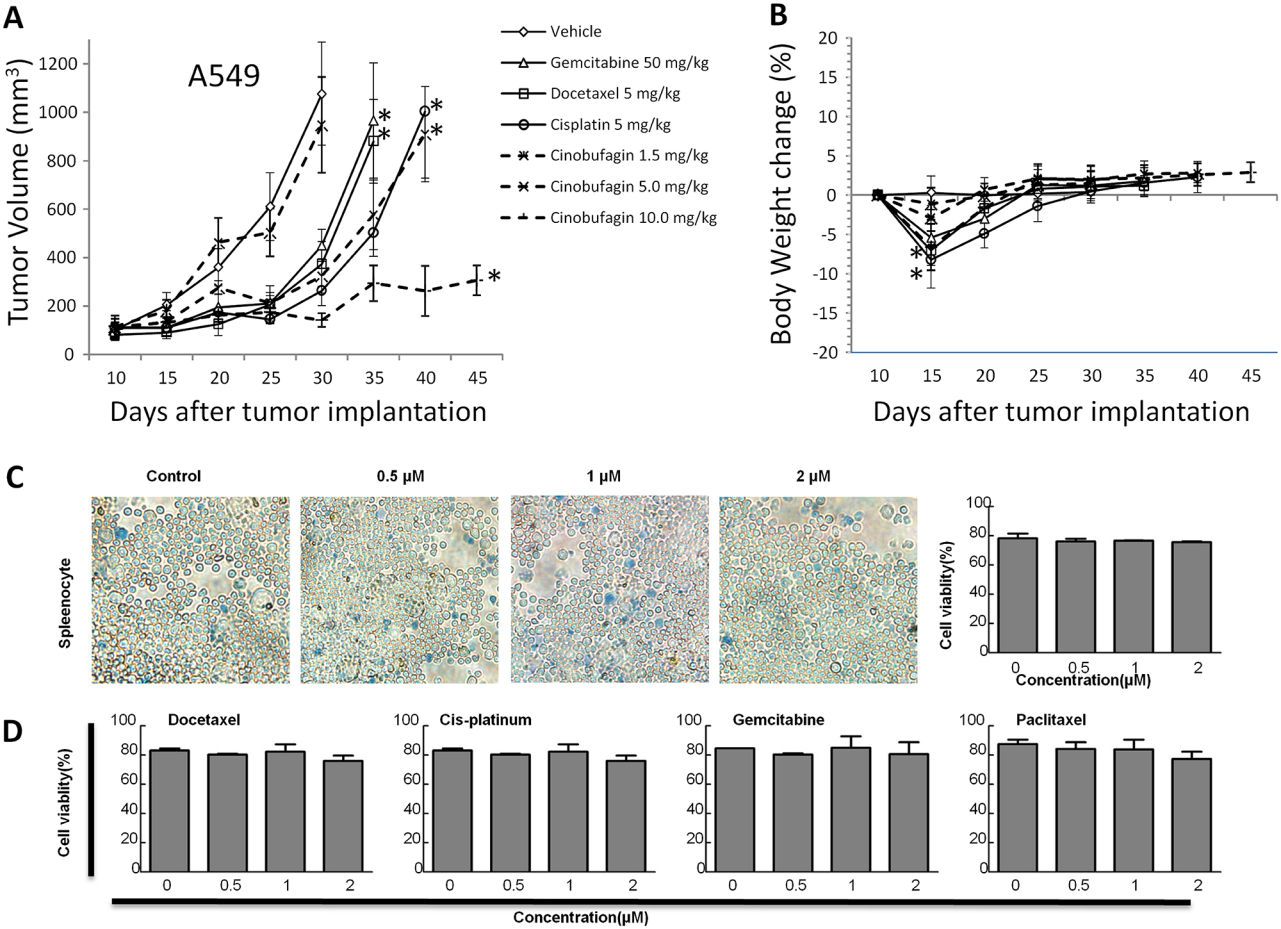
The effects of CB on *in vivo* tumor growth in human NSCLC cell lines and *in vitro* cell growth in rat splenocytes **A.** tumor volume and **B.** body weight over time in response to treatment with CB or platinum drugs in A549 NSG xenograft mouse models (n = 10 mice/each group). Error bars are SD. Down arrows indicate time point from each treatment with vehicle, CB or platinum drugs. All * *p* < 0.0001 vs. vehicle, Fisher's PLSD test. All experiments were repeated two times. **C** and **D.** rat splenocytes were treated for 24 hours with CB and platinum drugs, respectively. Representative images of the splenocytes with different concentrations of CB. Quantitative cell viabilities (%) are presented as mean ± SD of triplicates. All *p* > 0.05, one-way ANOVA followed by LSD test. All experiments were repeated three times.

To substantiate this observation, we treated the A549 cells with CB or platinum drugs in a NOD scid gamma (NSG) xenograft mouse model. Although treatment with a low dosage of CB (1.5 mg/kg/day) by intraperitoneal (IP) injection did not change xenograft tumor growth, there was significant inhibition of tumor growth in treatment with a middle dosage of CB (5 mg/kg/day), as compared to that from an effective dosage of platinum drugs (Figure 2A). Notably, the tumor growth was dramatically inhibited in treatment with high dosage of CB (10 mg/kg/day). The effect of CB or platinum drugs on body weight was also observed during the mice drug administration. The body weight was temporarily lost 5-10% at one week after administration (Figure 2B). Notably, the middle dosage of CB showed an anti-cancer efficacy with less than 5% body weight loss as compared to the other effective regimens. Furthermore, to investigate the cytotoxic effect of CB in normal cells, we isolated the splenocytes from one year-old rats. The cell viability was not significantly changed in rat splenocytes treated with 0.5, 1, and 2 μM CB (Figure 2C) or platinum drugs (Figure 2D), suggesting direct cytotoxicity of CB appears to be similar to that of platinum drugs in normal cells.

### CB induces the cell cycle arrest and apoptosis of human NSCLC cell lines

**Figure 3:**
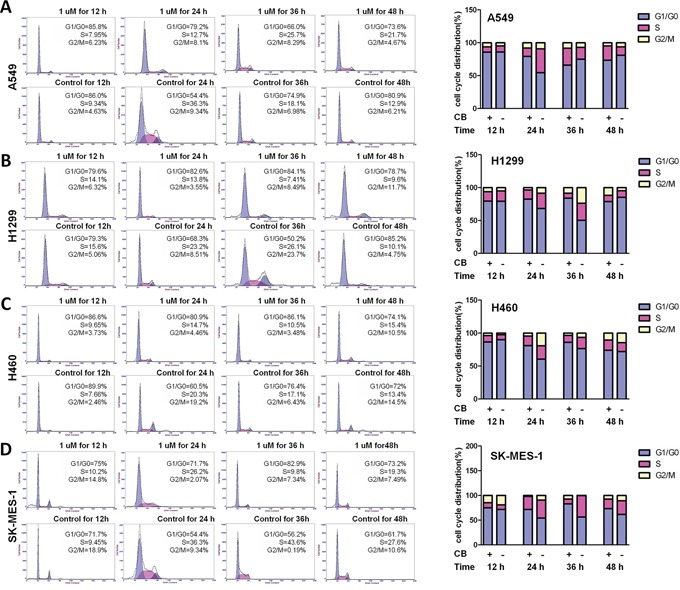
The effects of CB on cell cycle progression in human NSCLC cell lines Representative histograms represent the percent of different phases in cell cycle progression. After starvation for 48 hours, cell cycle progression in A459 **A.** H1299 **B.** H460 **C.** and SK-MES-1 **D.** cells were monitored by PI staining and flow cytometry at 12, 24, 36, and 48 hours after treatment with CB. Quantitative cell cycle progression for G1/G0, S, and G2/M phases are presented as percentage of cells in each cell cycle phase gate. All experiments were repeated three times.

Cell cycle arrest is one of the major causes of cell growth inhibition. Cinobufagin can induce cell cycle arrest and apoptosis in osteosarcoma cells [24]. To determine whether cell cycle arrest contributes to CB-induced cell growth inhibition in NSCLC cells, we observed the cell cycle progression during treatment with CB in four NSCLC cell lines using flow cytometry with propidium iodide (PI) staining. To observe the cell cycle progression, cells were arrested at the G0/G1 phase by serum starvation for 48 hours. As shown in Figure 3, after serum starvation, cell cycle was released by supplying serum, and more than 70% of CB-treated cells were arrested at G0/G1 phase at 24 hours. The cell cycle arrest was shown in a time-dependent manner and was also different in various cell types (Figure 3). After releasing for 24 hours, S phase was less in the CB-treated cells than in control cells, while for 24-48 hours the G2/M phase still remained in the CB-treated cells more than in control cells (Figure 3), indicating a significant cell cycle arrest by treatment with CB in human NSCLC cells.

**Figure 4:**
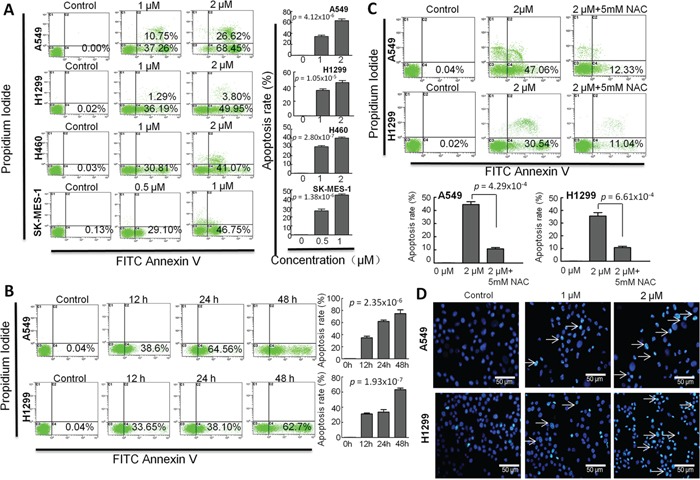
The effects of CB on apoptosis in human NSCLC cell lines The cell apoptosis was determined by flow cytometry with Annexin-V/PI staining. Representative histograms represent the percent of apoptotic or necrotic cells. The lower left quadrant (annexin V and PI negative) represents viable cells; The lower right quadrant (annexin V positive and PI negative) represents apoptotic cells in early stage; The upper left quadrant (annexin V negative and PI positive) represents necrotic cells or cellular debris; The upper right quadrant (annexin V and PI positive) represents late apoptotic or necrotic cells. **A.** A459, H1299 and H460 cells were treated with 0, 1 and 2 μM of CBfor 24 hours;SK-MES-1 cells were treated with 0, 0.5 and 1 μM of CB for 24 hours. **B.** cells were treated with CB for 0, 12, 24, 48 hours. **C.** cells were treated with CB at 0, 2 and 2 μM +5mM NAC for 24 hours. **D.** representative images observed by fluorescence microscopy represent the CB-induced apoptotic cells with Hoechst 33342 staining. White arrows indicate the apoptotic cells with fragmented nuclei. Quantitative apoptosis data are presented as the means and SD of triplicates. A and B *p* value, one-way ANOVA; C *p* value, two-tailed Student's *t* test. All experiments were repeated three times.

To determine the effect of CB on cell apoptosis in human NSCLC cell lines, we investigated both apoptosis and necrosis by flow cytometry with Annexin V/PI staining, and then observed nucleus morphology of apoptosis by fluorescence microscopy with Hoechst 33342 staining. As shown in Figure 4A, apoptotic cells were increased by treatment with CB at 24 hours in a dose-dependent manner in four NSCLC cell lines. Likewise, an increased apoptosis was also observed in a time-dependent manner following treatment with 1 μM CB for 48 hours in both A549 and H1299 cells (Figure 4B). Notably, the CB-induced apoptosis was significantly blocked when the cells were pretreated with 5mM antioxidant N-acetylcysteine (NAC) in both A549 and H1299 cells (Figure 4C). Since NAC can decrease mitochondrial-related oxidative stress [25], the CB-induced apoptosis may be involved in the intrinsic mitochondrial apoptosis [26–28]. Furthermore, DNA fragmentation is an important characteristic of apoptosis, which can be identified by Hoechst staining. Consistent with the above results, treatment with 1 and 2 μM of CB for 24 hours led to an observable increase of nuclear fragmentation in both A549 and H1299 cells (Figure 4D). This data suggests that CB-induced apoptosis may contribute to the cell growth inhibition of CB in NSCLC cells.

### Treatments of CB leads to the generation of reactive oxygen species (ROS) and disruption of the mitochondrial transmembrane potential (MMP) through an intrinsic mitochondrial apoptosis pathway in human NSCLC cell lines

**Figure 5:**
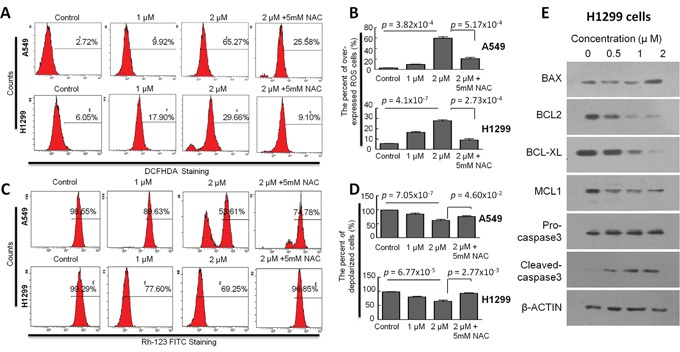
The effects of CB on ROS and MMP in human NSCLC cell lines **A** and **C.** representative histograms represent the levels of ROS (by DCFH-DA staining) and MMP (Rh-123 staining) in A549 and H1299 cells, respectively. **B** and **D.** quantitative ROS and MMP data are presented as the means and SD of triplicates in A549 and H1299 cells, respectively. All *p* value, Student's two-tailed *t* test or one-way ANOVA followed by LSD test. **E.** representative images for the expression of apoptotic related proteins determined by Western blot analysis after treatment with CB in H1299 cells.

To determine whether CB induces apoptosis through an intrinsic mitochondrial apoptosis pathway, we assessed the effect of CB on the ROS levels in both A549 and H1299 cells. Intracellular ROS generation in the cells was evaluated by flow cytometry with dichloro-dihydro-fluorescein diacetate (DCFH-DA) staining in both A549 and H1299 cells. As shown in Figure 5A and 5B, levels of ROS in the H1299 cells treated with 1 and 2μM CB were significantly increased from 6.05% to 17.90% and 29.66%, as compared to the control cells, and levels of ROS in A549 cells was significantly increased from 2.72% to 9.92% and 65.27%, as compared to the control cells. These effects were significantly blocked when the cells were pretreated with 5 mM NAC. Furthermore, since depolarization in the MMP is a characteristic feature of intrinsic apoptosis [29–33], the effect of CB on the MMP level was also examined by flow cytometry with Rhodamine (Rh)-123 staining in both A549 and H1299 cells. As shown in Figure 5C and 5D, levels of the MMP in H1299 cells treated with 1 and 2 μM of CB were significantly decreased from 99.29% to 77.60% and 69.25%, as compared to the control cells, and levels of the MMP in A549 cells treated with 1 and 2 μM of CB were significantly decreased from 98.55% to 89.63% and 53.61%, as compared to the control cells. Likewise, these effects were significantly blocked when the cells were pretreated with 5 mM NAC.

To elucidate the molecular mechanism underlying apoptosis induced by CB, the expressions of BCL-2 family members and related proteins, such as BCL-2, BCL-XL, BAX, MCL-1, and Caspase-3, were determined by Western blot in H1299 cells. As shown in Figure 5E, expression levels of BCL-2, BCL-XL, and MCL-1 were reduced by treatment with CB for 24 hours in a dose dependent manner, while expression level of BAX was increased in treatment with a high dosage of CB in H1299 cells. Although total Caspase-3 was not changed, cleaved-Caspase3 was increased by treatment with CB for 24 hours in a dose dependent manner (Figure 5E). These results together reveal CB-induced apoptosis through an intrinsic mitochondrial apoptosis pathway in NSCLC cells.

### CB inhibits AKT activation through a downregulation of both pAKT^T308^ and pAKT^S473^ in human NSCLC cell lines

**Figure 6:**
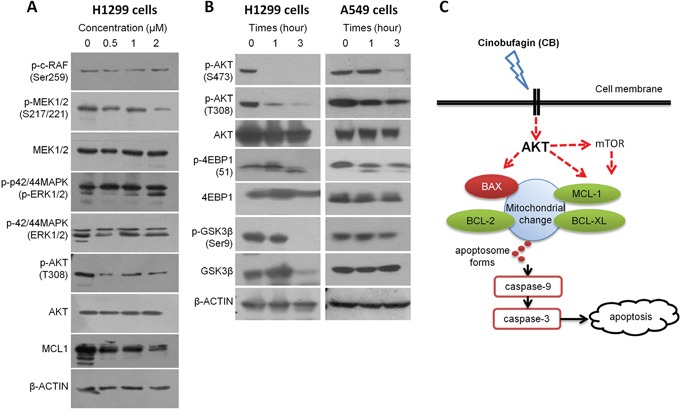
The effects of CB on RAF/MEK/ERK and PI3K/AKT/mTOR signaling in human NSCLC cell lines **A.** protein expression in RAF/MEK/ERK and AKT/MCL1 signaling determined by Western blot analysis after treatment with CB in H1299 cells. **B.** protein expression in AKT/mTOR and AKT/GSK3β signaling determined by Western blot analysis after treatment with CB in H1299 and A549 cells. All experiments were repeated three times. **C.** schematic representation of potential CB-induced apoptotic pathway in NSCLC cells.

Multiple signaling pathways are implicated in BCL-2 family-regulated caspase activation and apoptosis in cancer cells [34, 35]. Two major pathways, RAF/MEK/ERK and PI3K/AKT/mTOR signaling, play critical roles in tumorigenesis of NSCLC [36, 37], and targeting these two pathways can lead to BCL-2 family-related apoptosis in NSCLC cells [38–41]. Thus, we determined the potential effect of CB on these two signaling pathways in H1299 cells. As shown in Figure 6A, p-MEK1/2 appears to be decreased in the H1299 cells treated with CB for 24 hours, especially in a high dosage (2μM). However, p-c-RAF and pERK1/2 as upstream and downstream proteins of p-MEK1/2 were not changed in treatment with CB in H1299 cells, suggesting that RAF/MEK/ERK signaling is unlikely to be a major contributor for CB-induced apoptosis. Furthermore, we found that p-AKT^T308^ was dramatically downregulated by a low dosage (0.5μM) of CB in H1299 cells and this decrease was simultaneously associated with decreases of MCL-1. To validate this observation, we treated both H1299 and A549 cells with 1μM CB for 3 hours. As shown in Figure 6B, while expression levels of total AKT were not changed, both p-AKT^T308^ and p-AKT^S473^ were significantly reduced in a time dependent manner, suggesting that inhibition of AKT signaling may contribute to CB-induced intrinsic apoptosis in NSCLC cells. AKT is well known as a central node in signaling pathways consisting of many downstream components, such as mTOR, GSK-3β, etc. As expected, a critical downstream effector of mTOR signaling, p-4EBP1 was simultaneously downregulated with p-AKT after treatment with CB in both H1299 and A549 cells (Figure 6B), indicating a CB-induced inhibition of AKT/mTOR signaling. In addition, AKT directly stimulates the Ser9 phosphorylation of GSK-3β [42], resulting in the inactivation of GSK-3β, which can also inhibit MCL-1 [43]. Since the treatment with CB can simultaneously reduce p-AKT and MCL-1 (Figure 6A), we tested if downregulation of the MCL-1 by CB is through an AKT-GSK-3β axis. However, expression levels of p-GSK-3β at Ser9 were not changed after treatment with CB in both H1299 and A549 cells (Figure 6B), suggesting a GSK-3β-independent AKT-MCL-1 axis during CB-induced apoptosis in NSCLC cells.

## DISCUSSION

Here our data showed that CB effectively inhibits NSCLC cell growth in a dose-dependent manner as compared to platinum drugs, but this inhibition does not appear to be observed in rat splenocytes. Although slight tumor growth inhibition by a low dosage of CB (1.5 mg/kg) has been reported in a xenograft colon cancer model [44], we identified an effective dosage of CB (5 mg/kg or more) for tumor growth inhibition in a xenograft NSCLC model. Furthermore, we observed the CB-induced cell cycle arrest and apoptosis in NSCLC cells. Notably, we identified that CB inhibits tumor growth by inducing intrinsic mitochondrial apoptosis through AKT signaling pathway in NSCLC cells.

Cell cycle arrest and apoptosis have been considered as a main cause of cell growth inhibition [45]. Previous studies indicated that CB induces cell cycle arrest at the G2/ M phases in osteosarcoma cells [24], but at the S phase in breast cancer cells [46]. Our results indicate that treatment of NSCLC cells with CB induced cell cycle arrest in a time-dependent manner. However, we observed that, if cells release from the G0/G1 phases after serum starvation, CB induces cell cycle arrest, but this arrest is not aimed at a specific phase and is different in diverse NSCLC cell lines. Likewise, recent studies reported that CB induces apoptosis through a sequence of apoptotic modulators, including an increase of BAX, cytochrome C and caspase 3 and 9 in prostate cancer cells [19, 23], BAX/BCL-2 ratio in hepatocellular carcinoma cells [47], and BAX and cleaved-PARP in osteosarcoma cells [24], but a decrease of MCL-1 in several human cancer cell lines [48]. In the present study, we found that CB markedly induces apoptosis with an increase of BAX but a decrease of BCL-2, BCL-XL, and MCL-1 in NSCLC cells in a dose-dependent manner, suggesting that CB-induced apoptosis occurs through the intrinsic mitochondrial apoptosis pathway in NSCLC cells.

The regulation of intrinsic apoptosis by BCL-2 family proteins is through the raising of the cellular ROS level and subsequently reducing the MMP level, which stimulates mitochondria to release proapoptotic molecules, and results in the activation of caspase-9 and caspase-3 during apoptosis [50, 51]. In the present study, our data validated an increase of ROS and cleaved-caspase-3 and a decrease of MMP after treatment with CB, but they can be completely recovered by antioxidant NAC in NSCLC cells. Thus, crosstalk between BCL-2 family proteins, ROS, and MMP through an intrinsic mitochondrial apoptosis pathway is essential for understanding the anti-cancer mechanism of CB in NSCLC cells. In addition, targeting BCL-2 family proteins is an effective approach to inducing intrinsic apoptosis in cancer cells, which contributes to the cytotoxic therapies of chemotherapeutic drugs in NSCLC patients [34, 35]. Thus, CB may be an effective anti-cancer drug for NSCLC patients.

Comprehensive analysis of genetic alterations in NSCLC has identified key signaling pathways and genes, such as *EGFR*, *TP53*, *KRAS*, *PIK3CA*, *STK11*, *BRAF*, *ERBB2*, *MET*, and *PARK2* [34–37]. In the present study, we used 4 NSCLC cell lines (A549, H1299, H460, and SK-MES-1) that are harboring different genetic mutations in *EGFR*, *TP53*, *KRAS*, *NRAS*, *PIK3CA*, *STK11*, *CDKN2A*, etc. Thus, CB-induced intrinsic mitochondrial apoptosis as well as cell cycle arrest may occur through those key signaling pathways in NSCLC cells. In fact, recent studies reported that CB suppresses cell proliferation and induces cell cycle arrest and apoptosis through downregulation of p-ERK, c-MYC, p-GSK-3β, NK-κB/p65, and p38 MAPK, but upregulation of intracellular free calcium ([Ca2+]i) and Cyclin A in cancer cells [19, 24, 46, 47, 52–54]. In the present study, we observed that treatment with CB dramatically reduced both p-AKT^T308^ and p-AKT^S473^ in H1299 and A549 cells. Interestingly, downregulation of p-AKT by treatment with CB was always accompanied with the same trend reduction of p-4EBP1 and MCL-1 but not p-GSK-3β, suggesting CB-induced AKT/mTOR apoptotic signaling in NSCLC cells (Figure 6C) [55–59].

In conclusion, our findings reveal that CB markedly inhibited tumor growth and induced cell cycle arrest and apoptosis in human NSCLC cells. Our results suggest that CB through AKT signaling pathway and the crosstalk between BCL-2 family proteins, ROS, and MMP leads to an intrinsic mitochondrial apoptosis (Figure 6C). CB may be a potential alternative anti-cancer drug to platinum drugs for anti-lung cancer therapy.

## MATERIALS AND METHODS

### Cell lines, antibodies, and reagents

Human NSCLC cell lines, including human lung adenocarcinoma cell line A549, human lung adenocarcinoma cell line H1299, human large cell lung carcinoma cell line H460, and lung squamous carcinoma cell line SK-MES-1, were purchased from Cell Bank of Chinese Academy of Sciences (Shanghai, China). A549, H1299 and H460 cells were cultured in Roswell Park Memorial Institute Medium (RPMI 1640) in a medium supplemented with 10% fetal bovine serum (FBS). SK-MES-1 cells were cultured in Dulbecco's Modified Eagle's Medium (DMEM) in a medium supplemented with 10% FBS and maintained at 37°C with 5% CO_2_ in a humidified atmosphere.

Cinobufagin (CB) which was purchased from the Chinese materials research center (Beijing, China), was dissolved in Dimethyl Sulfoxide (DMSO) purchased from Shenggong Company (Shanghai, China) to make a stock solution. FBS was purchased from Gibco (Grand Island, NY, USA). DMEM, RPMI-1640, 3-(4,5-Dimethylthiazol-2-yl)-2,5-Diphenyltetrazolium Bromide (MTT), Hoechst 33342, and Rh-123 mitochondrial specific fluorescent dye were purchased from Sigma (Shanghai, China). Cell Cycle Analysis Kit and Reactive Oxygen Species Dichlorofluorescin-Diacetate (DCF-DA) Detection Kit were purchased from Beyotime Company (Shanghai, China). BCA Protein Assay Kit and Annexin V-FITC Apoptosis Detection Kit were purchased from Keygen Company (Nanjing, China). Polyclonal antibodies against β-ACTIN, BAX, BCL-2, BCL-XL, MCL-1, AKT, p-AKT^T308^, p-AKT^S473^, GSK3β, p-GSK3β^Ser9^, p-c-RAF^Ser259^, MEK1/2, p-MEK1/2^S217/221^, p42/44MAPK (ERK1/2), p-p42/44MAPK (p-ERK1/2), TSC1, TSC2, 4EBP1, p-4EBP1, pro-caspase-3, cleaved-caspase3, and horseradish peroxidase-conjugated secondary antibodies (goat-anti rabbit and mouse) were purchased from Cell Signaling (Shanghai, China). Western Blotting Detection Kit was purchased from Milipore (Billerica, USA).

### Cell growth inhibition assay

The inhibition of cell growth was determined by an MTT assay. Briefly, four NSCLC cells were treated with various concentrations of CB and platinum drugs, including cisplatin, gemcitabine, docetaxel, and paclitaxel for 24 hours. Following treatment, the MTT reagent was added (100 μl/ml) and the cells were further incubated at 37°C for 4 hours. Then, 150 μl DMSO was added to dissolve the formazan crystals and absorbance was read in a micro-plate reader (Thermo Scientific, Waltham, MA, USA) at 570 nm. The viable cell number was directly proportional to the production of formazan. The growth inhibition assay was repeated three times. The IC_50_ values were calculated using GraphPad Prism 5 (La Jolla, CA, USA). The percentage of inhibition was calculated as follows: Inhibitory ratio (%)=(A[control]–A[sample])/A[control]×100%.

### Xenogeneic transplantation

NSG mice were purchased from The Jackson Laboratory (Bar Harbor, USA). The male F1 mice of given genotypes were used in the study. All animal experiments were conducted in accordance with accepted standards of animal care and approved by the Institutional Animal Care and Use Committee of The Second Hospital of Jilin University. The A549 cells were grown in DMEM media supplemented with 10% FBS without antibiotics. Cells were expanded and used for inoculation within three to four passages after thawing from liquid nitrogen storage. Eight week old male NSG mice were used as recipients to received subcutaneous injections of 100 μL containing 5 × 10^6^ A549 cells in 50:50 Matrigel/collagen I on their left flank. Ten days after injection, the mice were randomized into seven groups (n = 10 mice/group) for the following treatments: vehicle control, cisplatin (5 mg/kg/day, every 5 days), gemcitabine (50 mg/kg/day, every 5 days), docetaxel (5 mg/kg/day, every 5 days), paclitaxel (5 mg/kg/day, every 5 days), and CB (1.5, 5.0, and 10.0 mg/kg/day, respectively, every 5 days). Tumor size was measured using a Vernier caliper every day in the two longest dimensions. Tumor volume was calculated with the following formula 0.5D1(D2)^2^, where D1 is the long measure and D2 is the short dimension. Mouse body weight was measured every 5 days until the study end. Each mouse was euthanized when tumor size reached 1,500 mm^3^, when the tumor became ulcerated, or when tumor growth was observed up to 45 days after tumor implantation.

### Cell cycle progression assay

For cell cycle analysis by flow cytometry, we used PI Cell Cycle Detection Kit. Briefly, four NSCLC cells were serum starved for 48 hours and then treated with CB at the concentration of 0, 1, 2 μM, respectively. After 12, 24, 36, and 48 hours of treatment with CB, cells were harvested and fixed in 500 μl 70% ice cold ethanol at 4°C for 2 hours. Next, the samples were washed with phosphate buffer saline (PBS) and incubated with RNase A and PI staining solution as the manufacturer described. After staining, the samples were analyzed by flow cytometry.

### Apoptosis detection assay

Four NSCLC cells were treated with CB at various concentrations and various times (0, 12, 24, and 48 hours) according to the MTT assay. Cells were collected and washed with PBS, and then the apoptotic cell death rate was examined by Annexin V-FITC and PI double staining using the Annexin V-FITC Apoptosis Detection Kit according to the manufacturer's instructions. After staining with Annexin V-FITC/PI, the samples were analyzed by flow cytometry (Beckman Coulter Epics XL, Brea CA, USA). A549 and H1299 cells were treated with CB in a dose dependent manner in the presence or absence of 5 mM NAC.

To visualize apoptotic cell death and nuclear morphology, cells were stained with Hoechst. Briefly, cells were seeded into a 6-well flat bottom plate and treated with CB at the concentrations of 0, 1, 2 μM respectively. After 24 hours of treatment, cells were collected, washed and allowed to dry on slides. Then nuclei were stained with Hoechst for 10 min. The apoptotic cells displaying fragmented or condensed nuclei were observed under a fluorescence microscope (Olympus, Tokyo, Japan).

### Determination of ROS and MMP

ROS level was measured in A549 and H1299 cells by using a DCF-DA Detection Kit according to the manufacturer's instructions. Briefly, cells were treated with CB at the concentrations of 0, 1, 2 μM, respectively, in the presence or absence of 5 mM NAC. After 24 hours of treatment, cells were collected, washed with RPMI-1640 without FBS, and then incubated with 10mM DCF-DA at 37°C for 15 minutes. The stained cells were washed and resuspended in 200 μl RPMI-1640. The intracellular ROS activated the oxidation of DCFH to the fluorescent compound DCF. The generation of ROS was then analyzed by flow cytometry.

Mitochondrial transmembrane potential was determined in A459 and H1299 cells by flow cytometry with Rh-123 staining according to the manufacturer's instructions. Briefly, cells were treated with CB at the concentrations of 0, 1, 2 μM, respectively, in the presence or absence of 5 mM NAC. After 24 hours of treatment, cells were collected, washed with PBS and then incubated with Rh-123 (10 μM) at 37°C for 20 minutes. The stained cells were washed and resuspended in 200 μl PBS. The level of mitochondrial transmembrane potential was then analyzed by flow cytometry.

### Western blot analysis

To determine the mechanism underlying the CB-induced apoptosis, western blot was performed for apoptotic related proteins. Firstly, H1299 cells were treated with CB at the concentrations of 0, 0.5, 1, 2 μM, respectively. H1299 cells and A549 cells were also treated with 1μM of CB after 1, 3, and 6 hours, cells were collected and washed with PBS. The cell pellets were resuspended in Radio-Immunoprecipitation Assay (RIPA) lysis buffer and ultrasound lysed on ice. After centrifugation, the supernatant fluids were collected and the protein contents of the supernatant were determined using BCA Protein Assay Kit before the protein samples were stored at −20°C. The protein lysates were then separated by electrophoresis on 10% sodium dodecyl sulphate-polyacrylamide gel and transferred to a polyvinylidene fluoride membrane (Amersham Biosciences, Piscataway, NJ, USA). The membranes were soaked in a blocking buffer (5% skimmed milk) for 2 hours. To probe for all the proteins, membranes were incubated overnight at 4°C with relevant antibodies, followed by appropriate horseradish peroxidase conjugated secondary antibodies and enhanced chemiluminescence detection. We used the Gel-Pro Analyzer, which could extract valuable qualitative and quantitative information from electrophoretic gels to document and store our Western blot data.

### Statistical analysis

All data represent at least three independent experiments and were expressed as Mean ± standard deviation (SD). The means of the variables were compared using a two-tailed *t* test between two groups. Multiple comparisons were made using a one-way ANOVA followed by least significant difference (LSD) test. The mean of one group was compared with the mean of another group by Fisher's protected LSD (PLSD, a two-ways ANOVA) test. All data were entered into an access database using Excel 2010 and analyzed with SPSS (version 20; IBM, Armonk, NY, USA) and StatView (version 5.0.1; SAS Institute Inc., Cary, NC, USA). *P* < 0.05 was considered statistically significant.
